# Genome sequencing reveals novel causative structural and single nucleotide variants in Pakistani families with congenital hypogonadotropic hypogonadism

**DOI:** 10.1186/s12864-024-10598-3

**Published:** 2024-08-14

**Authors:** Yassine Zouaghi, Anbreen Mazhar Choudhary, Saba Irshad, Michela Adamo, Khaleeq ur Rehman, Ambrin Fatima, Mariam Shahid, Nida Najmi, Fernanda De Azevedo Correa, Imen Habibi, Alexia Boizot, Nicolas J. Niederländer, Muhammad Ansar, Federico Santoni, James Acierno, Nelly Pitteloud

**Affiliations:** 1 University of Lausanne, Lausanne, Switzerland; 2 Service of Endocrinology, Diabetology and Metabolism, Lausanne University Hospital, Avenue de La Sallaz 8, Lausanne, CH-1011 Switzerland; 3 School of Biochemistry and Biotechnology, University of the Punjab, Lahore, Pakistan; 4 FMH College of Medicine & Dentistry, Lahore, Pakistan; 5 Department of Biological and Biomedical Sciences, Aga Khan University, Karachi, Pakistan; 6 Centre of Excellence in Molecular Biology, University of the Punjab, Lahore, Pakistan; 7 Department of Obstetrics and Gynaecology, The Aga Khan University Hospital, Karachi, Pakistan; 8 Department of Ophthalmology, University of Lausanne, Jules Gonin Eye Hospital, Fondation Asile Des Aveugles, Lausanne, Switzerland; 9 Advanced Molecular Genetics and Genomics Disease Research and Treatment Centre, Dow University of Health Sciences, Karachi, Pakistan; 10 Medigenome, Geneva, Switzerland

**Keywords:** Congenital hypogonadotropic hypogonadism, Whole genome sequencing, Copy number variants, Rare endocrine disease, Infertility

## Abstract

**Background/Objectives:**

This study aims to elucidate the genetic causes of congenital hypogonadotropic hypogonadism (CHH), a rare genetic disorder resulting in GnRH deficiency, in six families from Pakistan.

**Methods:**

Eighteen DNA samples from six families underwent genome sequencing followed by standard evaluation for pathogenic single nucleotide variants (SNVs) and small indels. All families were subsequently analyzed for pathogenic copy number variants (CNVs) using CoverageMaster.

**Results:**

Novel pathogenic homozygous SNVs in known CHH genes were identified in four families: two families with variants in *GNRHR*, and two others harboring *KISS1R* variants. Subsequent investigation of CNVs in the remaining two families identified novel unique large deletions in *ANOS1*.

**Conclusion:**

A combined, systematic analysis of single nucleotide and CNVs helps to improve the diagnostic yield for variants in patients with CHH.

**Supplementary Information:**

The online version contains supplementary material available at 10.1186/s12864-024-10598-3.

## Introduction

Congenital hypogonadotropic hypogonadism (CHH) is a rare genetic endocrine disorder resulting in partial or absent puberty and infertility due to defects in gonadotropin-releasing hormone (GnRH) secretion and/or action. The frequency of CHH is estimated to be between 1:86,000 [1] and 1:10,000 [2], and a reported male predominance from 5:1 [3] to 2:1 [4]. Clinically, CHH is difficult to distinguish from constitutional delay of growth and puberty (CDGP) during early adolescence, but the presence of micropenis and/or cryptorchidism in male neonates may be early clues for long-term GnRH deficiency. The clinical diagnosis of CHH is primarily a diagnosis of exclusion after other common causes of hypogonadotropic hypogonadism have been ruled out [5]. Approximately 50% of CHH patients present with anosmia – Kallmann Syndrome – due to defects affecting the olfactory and GnRH neuron systems during fetal development [6].

Pathogenic variants in more than 65 genes [5, 7, 8] have been associated with both non-syndromic and syndromic CHH. While these variants are present in up to 50% of cases [9], these variants only fully explain the CHH phenotype in closer to 25% of patients [10]. Previous studies focused primarily on patients from European descent [1, 2], however one recent study found a similar frequency of genetic variants in a non-European population [11]. Dominant, recessive, X-linked inheritance, and uniparental disomy (UPD) have been observed in CHH families, and variable expressivity and incomplete penetrance are prevalent in this disorder [12, 13]. Furthermore, the rapid discovery of new CHH genes coupled with advances in high-throughput sequencing (HTS), namely whole exome sequencing (WES) and genome sequencing (WGS), have increased our genetic understanding of CHH and uncovered a notable number of CHH patients with oligogenic inheritance [14].

Several of the known CHH genes were discovered through the detection of copy number variants (CNVs—large insertion/deletion variants), most notably *ANOS1* [15] and *FGFR1*, [16] among others. Despite this, routine screening evaluation of CHH genes for CNVs is lacking, primarily due to the high cost and time for traditional assays such as karyotyping, fluorescence in situ hybridization (FISH), multiplex ligation-dependent probe amplification (MLPA) or array comparative genomic hybridization (CGH). Although HTS has been available for over a decade, recent advances in bioinformatic analysis of HTS data demonstrated its value to detect CNVs [17].

The current study evaluates six families segregating CHH and originating from remote areas of Punjab, Pakistan—an underrepresented population in the genetic studies of this disorder. Using a combination of traditional single nucleotide variants (SNVs) and advances in CNV detection, we successfully determined the underlying genetic causes of CHH in all families.

## Results

### Clinical findings

**Fig. 1:**
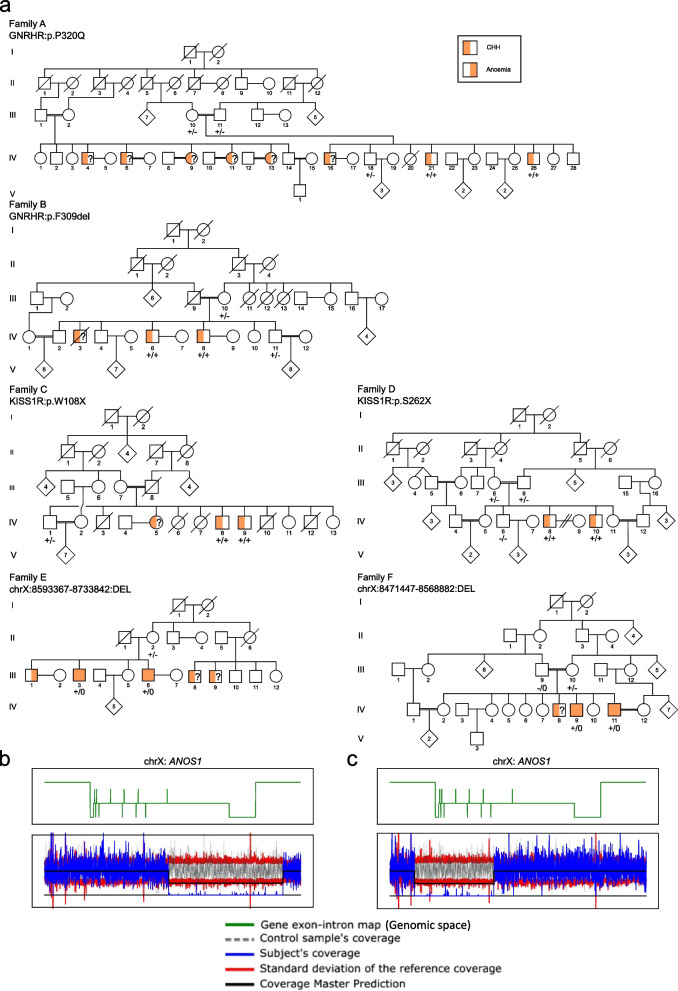
Consanguineous families’ pedigrees and CNVs detected in *ANOS1*. **a** Pedigrees of the 6 consanguineous families. **b** Output of CoverageMaster on WGS for the region around *ANOS1* in Subject III:6. The top panel shows the exon–intron map of *ANOS1* in the genomic space. The bottom panels show the coverage for subject III:6 compared to the sequencing batch and controls in the genomic space. **c** Output of CoverageMaster on WGS for the region around *ANOS1* in Subject IV:9. The top panel shows the exon–intron map of *ANOS1* in the genomic space. The bottom panels show the coverage for subject IV:9 compared to the sequencing batch and controls in the genomic space

We present six families (A-F) with 24 affected individuals (20 males and 4 females) of which 12 CHH men have been studied. All participants were evaluated at multiple stages of their development, and the final diagnosis was given after a detailed medical consultation and hormonal blood tests at 18 years old or later to confirm the absence of pubertal development. Eight participants were normosmic CHH (nCHH) and four were hyposmic (KS). All presented with prepubertal testicular volume (< 3 mL), and one patient had a history of cryptorchidism (Family C, V-8). No hypothalamic or pituitary anomalies were detected on brain MRI. No other CHH-associated phenotypes (e.g. renal agenesis, synkinesia, etc.) were observed in our study population. The clinical evaluations of the participants are summarized in Table 1 and Fig. 1a. All unaffected family members had normal hormonal evaluations, and no defects in smell and/or fertility.

### SNV analysis

Homozygous pathogenic variants in *GNRHR* were identified in affected individuals of Families A and B (Fig. 1a, Table 1). All affected members were normosmic, which is consistent with a defect in GnRH action at the pituitary level. Family A is a large consanguineous pedigree including 8 affected individuals with nCHH (5 males and 3 females). DNA was available on two affected males (21.IV & 26.IV), their unaffected brother (18.IV) and parents (10.III & 11.III). A novel homozygous p.[Pro320Gln];[Pro320Gln] variant was identified in both affected patients in Family A (Fig. 1a). This variant is absent from gnomAD, has a CADD of 27.5, and predicted as damaging by SIFT. Additionally, an alternative variant at the same position results in binding defects (with GnRH), as shown in transiently transfected cells resulting in a total loss of function [18]. Consequently, p.Pro320Gln meets the PP3, PM5, PM2, PP2 and is classified as likely pathogenic according to ACMG standards. Family B also resulted from consanguineous marriages. Three brothers are affected with nCHH. DNA analyses were performed on two affected brothers (6.IV & 8.IV), one unaffected brother (11.IV) and the unaffected mother (10.III) Similarly, all affected patients in Family B harbored homozygous p.[Phe309del];[Phe309del] *GNRHR* variants (MAF = 6.72 × 10–5 in gnomAD with no homozygotes observed). This variant is predicted to be damaging with SIFT, has a CADD of 21.8, meaning it is within the top 0.66% of the most pathogenic variants in humans, and co-segregates perfectly with the CHH phenotype. This variant meets the PM4, PM2 and PP1 strong ACMG criteria leading to a likely pathogenic classification.

**Table 1:**
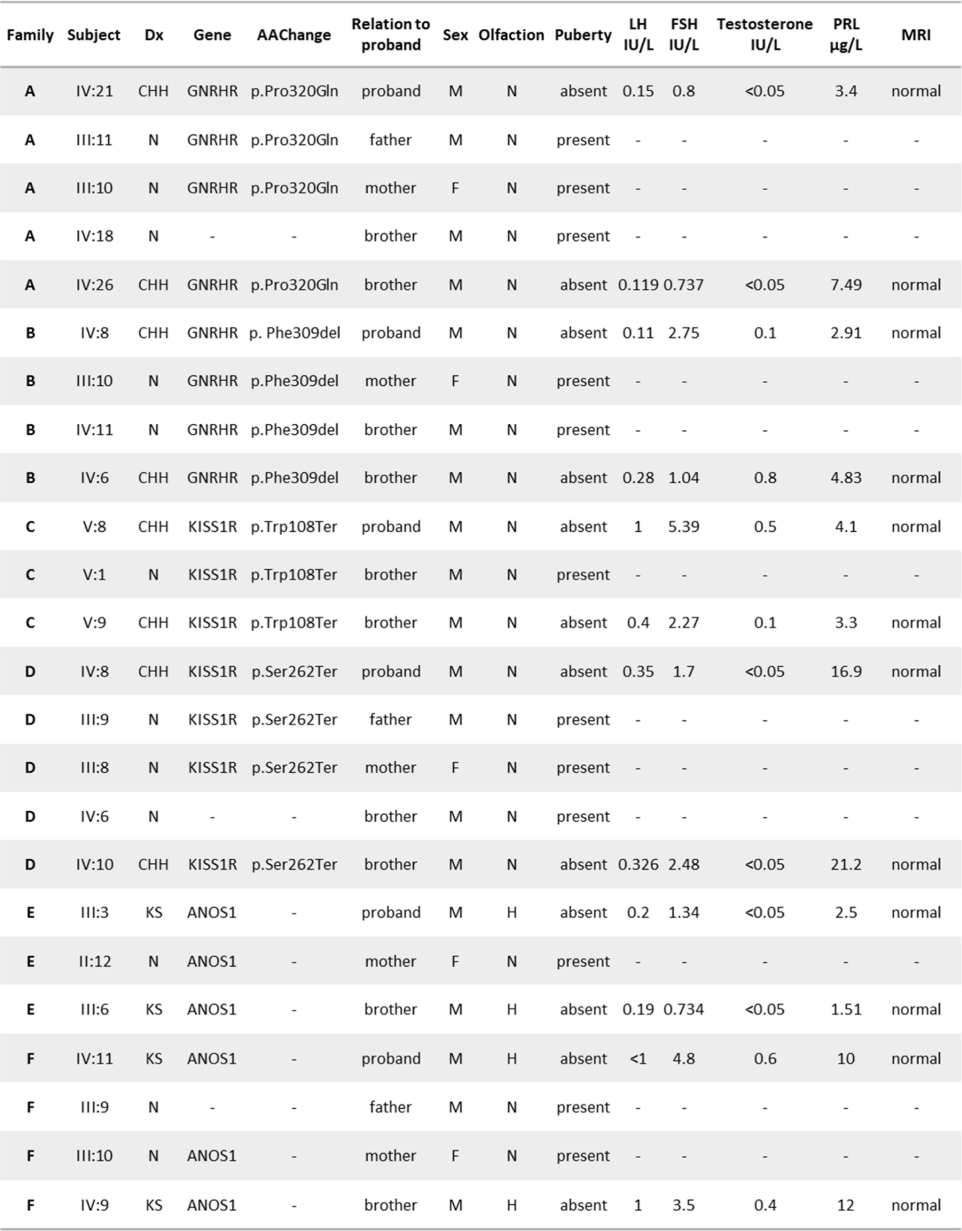
Families A-F clinical information

Variants found in families A-F in CHH genes with clinical information on their members. *DX* Diagnosis,  *AAChange* Amino acid change, *N* Normal phenotype, *M* Male, * F *Female, *A* Anosmic

Homozygous truncating variants in *KISS1R* were identified in the affected individuals evaluated in both Families C and D, consistent with the autosomal recessive inheritance mode. Family C is segregating a p.[Trp108Ter];[Trp108Ter] variant, while Family D is segregating a p.[Ser262Ter];[Ser262Ter] variant. Neither of these variants is found in gnomAD nor in Clinvar [19], and both are considered pathogenic by ACMG classification due to their likely undergoing nonsense mediated decay (NMD) resulting in complete loss-of-function.

The SNVs observed in Families A-D were confirmed by Sanger sequencing in all available samples, and segregation analysis was consistent with their involvement. The homozygous nature of the pathogenic variants in all four families (Families A-D) is consistent with the consanguineous matings present. No additional SNVs in CHH genes were found in the affected individuals from Families A-D. Furthermore, no putative causative SNVs in CHH genes were identified in Families E and F.

### CNV analysis

As expected given the SNV results, no relevant CNVs were detected in CHH genes in Families A-D. However, hemizygous CNVs were present in *ANOS1* on chromosome X in Family E and F (Fig. 1a,b and Table 1). In Family E, a 140 kb deletion beginning 33 kb upstream of *ANOS1* and extending through the first exon and intron of the gene was detected. A 100 kb deletion was also observed in Family F and encompassed the last 11 of the 14 exons of *ANOS1*. Similar CNVs were absent in both DGV and gnomAD, and are considered pathogenic according to ACMG classification [20] given their truncating nature. The segregation of these pathogenic *ANOS1* variants in both families is consistent with the known X-linked mode of inheritance for this gene. All male affected family members tested for olfactory defects have CHH with anosmia/hyposmia, also known as Kallmann syndrome (Fig. 1, Table 2).

**Table 2:**
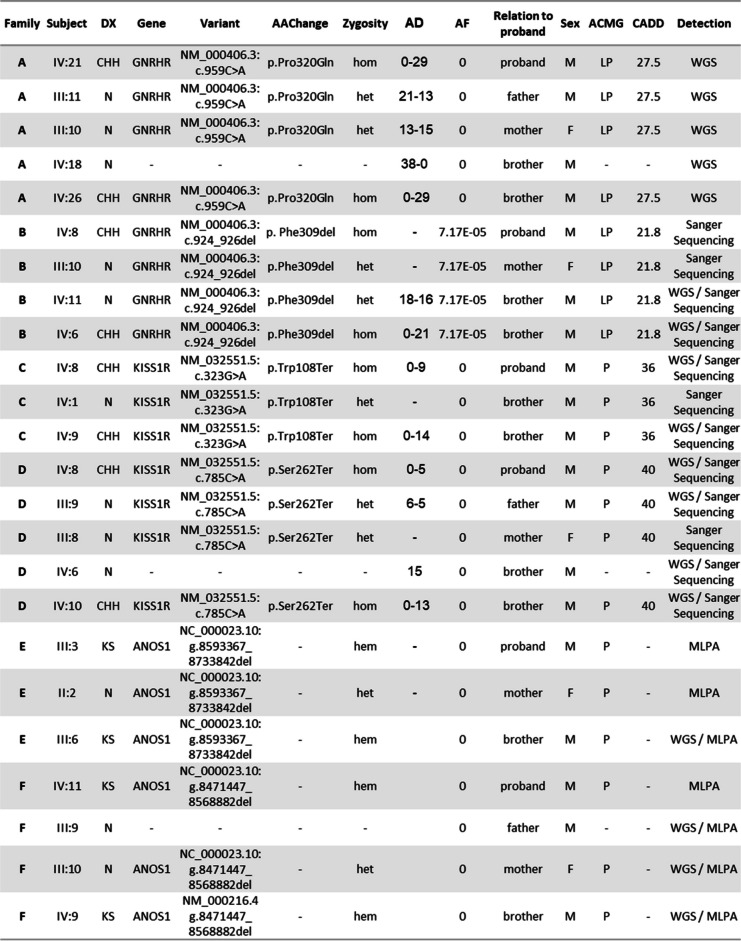
Families A-F CHH segregating variants

Variants found in families A-F in CHH genes and their description. *DX* Diagnosis, KS* Suspected KS, *N *Normal phenotype, *AAChange* Amino acid change, *hom* homozygous, *het *heterozygous, *hem *hemizygous, *M *Male, *F *Female, *LP* Likely pathogenic, *P* Pathogenic

## Discussion

Previous studies have demonstrated that pathogenic SNVs and small insertion/deletion variants can be found in up to 50% of CHH patients [7, 9, 21] However, the diagnostic yield is much lower [10]. The current study uncovered novel pathogenic or likely pathogenic variants in known CHH genes that explain the patients’ phenotype in all 6 families. One key factor of this diagnostic success is the use of Whole Genome Sequencing (WGS) to subsequently evaluate patient DNA for Copy Number Variations (CNVs) when standard Single Nucleotide Variant (SNV) analysis is negative. It’s important to note that CNVs are better detected in WGS compared to Whole Exome Sequencing (WES), making WGS a more effective tool for comprehensive genetic investigation [22, 23]. This is particularly useful when further investigation is needed to find genetic causes for CHH. CNVs were evaluated in two prior CHH studies, the first one utilizing the relatively expensive and unscalable MLPA method [24], and the second [25] using WES data with 7.4% and 2% diagnostic yield, respectively. It’s worth noting that increased diagnostic yield has been observed in other diseases as well when using similar genomic analysis techniques. This underscores the broad utility and effectiveness of these methods in enhancing our understanding of various diseases [17, 26, 27]. As shown in our study and many others, the advent of WGS and its rapidly decreasing cost now allows for more efficient and productive evaluation of CNVs in the increasing number of CHH-associated genes in both exonic and intronic regions.

Intriguingly, two Pakistani families harbored novel homozygous pathogenic variants in *KISS1R*—a notable finding since the frequency of *KISS1R* variants in CHH patients is quite rare (< 1.0%) [10]. To date, only nine loss-of-function variants have been described in this gene [28] (Fig. S1) since its discovery two decades ago [29]. KISS1R-deficient individuals are normosmic and exhibit severe GnRH deficiency. This is in line with the crucial role of the galanin-like G protein-coupled receptor encoded by KISS1R on the regulation of GnRH secretion. The patients’ symptoms are consistent with those previously reported for *KISS1R* variants [7]. Two families harbored homozygous missense variants in *GNRHR*, the first gene identified to cause nCHH in 1997 [30] and underlying 4% of CHH cases [31]. One of them, p.Pro320Gln, is not found in any control databases. *GNRHR* encodes for the type 1 GnRH receptor, GnRHR, a G protein-coupled receptor expressed in the gonadotrophs. Natural GnRHR mutants are frequently recognized by the cellular quality control system as misfolded and are retained in the endoplasmic reticulum [32, 33]. Of interest, pharmacochaperones rescue misfolded Gnrhr in murine models, disable the ability of Gnrhr mutants to reach the plasma membrane, and restore their ability to respond to endogenous Gnrh ligand, thus being a promising strategy to treat CHH patients with a genetic profile similar to the affected individuals in Family A [34].

It is important to remark that the genetic causes of CHH have been widely studied in patients mainly from European and North American populations. Indeed, only one publication has specifically evaluated CHH genes in a single Pakistani family [35]. The current manuscript not only demonstrates once more the improved diagnostic potential of WGS over the classical hybridisation-based sequencing methods, but also highlights the diagnostic utility of medical genetics in under-represented populations.

## Materials and methods

### Patients

This study included 24 individuals from six Pakistani families with at least two members affected by CHH. Five of the families reported consanguinity. Patients were diagnosed with CHH in accordance with the guidelines presented in the European Consensus Statement on CHH [5]. In short, CHH diagnosis included: (1) absent or partial puberty by age 17 years, (2) low or normal gonadotropin levels in the context of low serum levels of sex steroids (testosterone or estradiol), (3) a normal hypothalamus and pituitary on imaging, and (4) otherwise normal anterior pituitary function [5]. Smell tests were performed using UPSIT [36].

### DNA extraction and sequencing

DNA from the 24 participating family members was extracted in Pakistan. Eighteen of the samples had DNA of sufficient quality for WGS sequencing. DNA from the remaining seven individuals was retained for subsequent SNV or CNV confirmation (see SNV and CNV analysis, below) once a pathogenic variant was found to be segregating in the family. Paired-end WGS was performed using the DNBSEQ technology through the Denmark facility of BGI (Beijing Genomics Institute) Global. The DNBs (DNA nanoballs) were loaded into a patterned nanoarray, and paired-end reads of 100–150 bases were generated by probe-anchor synthesis (cPAS). Each sample was sequenced to a minimal depth of 30X.

The resulting raw sequences (BGI fastq files) were processed by an in-house bioinformatics analysis workflow which relies on Sentieon DNASeq (v202112.05), a GATK compliant toolbox [37, 38] that maps the reads to the human reference sequence (GRCh37) and detects SNVs and short insertions/deletions (Indels), smaller than 50 bp. Identified variants were then annotated with minor allele frequencies (MAFs) from gnomAD (v2.1.1) [39] and with multiple pathogenicity prediction techniques including CADD (v1.6) [40] and SpliceAI (v1.3) [41] using ANNOVAR (v2020-06–07) [42].

### SNV and CNV analysis

Selected variants satisfied at least one of the following criteria: nonsense (stop gain, frameshift, and acceptor–donor splice sites ± 2 bp from an exon), missense, inframe indels, and variants with a probability higher than 0.8 of causing a splicing defect as determined by the SpliceAI algorithm [41].

All the variants present in one of 65 CHH genes (see Supplementary Table S1) passed GATK filters, including a minimum quality score (QS) of 50. In line with inheritance patterns observed in rare diseases, variants with a minor allele frequency (MAF) of less than 1% were deemed potentially pathogenic if homozygous, and those with a MAF of less than 0.01% if heterozygous. Variants passing these filters were further annotated using Varsome [43] for classification of pathogenicity according to the American College of Medical Genetics (ACMG) standards. Sanger sequencing was used to confirm and evaluate segregation in all families, including members not sent for WGS.

CoverageMaster (v1.0) [44] was used to detect CNVs in CHH genes. In brief, this program uses depth of coverage from WGS or WES, and compresses these data into a multiscale wavelet space. The output is then analyzed through an iterative Hidden Markov Model to detect insertions or deletions > 50 bp at nucleotide-scale resolution. In addition, 30 unrelated samples sequenced with the same technology were used as controls for the CoverageMaster analysis. This helps to identify errors of sequencing/assembly and frequent CNVs. The Database of Genomic Variants [45] and gnomAD for structural variants [46] were also used as controls. MLPA was used to confirm CNVs in all available DNA samples. For *ANOS1*, the SALSA MLPA probemix P132-A4 kallmann-1 kit (MRC Holland) was used according to the manufacturer’s protocol to validate the variants.

### Supplementary Information

Supplementary Material 1.

Supplementary Material 2.

## Data Availability

The genetic data pertinent to this study, specifically the BAM files, have been deposited in the European Nucleotide Archive (ENA). The project can be accessed using the accession number PRJEB76310. Individual samples within this project are identifiable with unique accession numbers, which range from ERR13194595 to ERR13194658. The nomenclature for the sample names aligns with the identities presented in this article, adhering to the format: family_generation_individual. For further details or queries, please reach out to the corresponding author, Nelly Pitteloud.
