# Supplementary material for: Genome sequencing reveals novel causative structural and single nucleotide variants in Pakistani families with congenital hypogonadotropic hypogonadism
Source: BMC Genomics. 2024 Aug 14;25:787. doi: 10.1186/s12864-024-10598-3 (PMC11325732; doi:10.1186/s12864-024-10598-3)
Supplement: Supplementary file 1 — Supplementary Material 1. [file 12864_2024_10598_MOESM1_ESM.pdf]

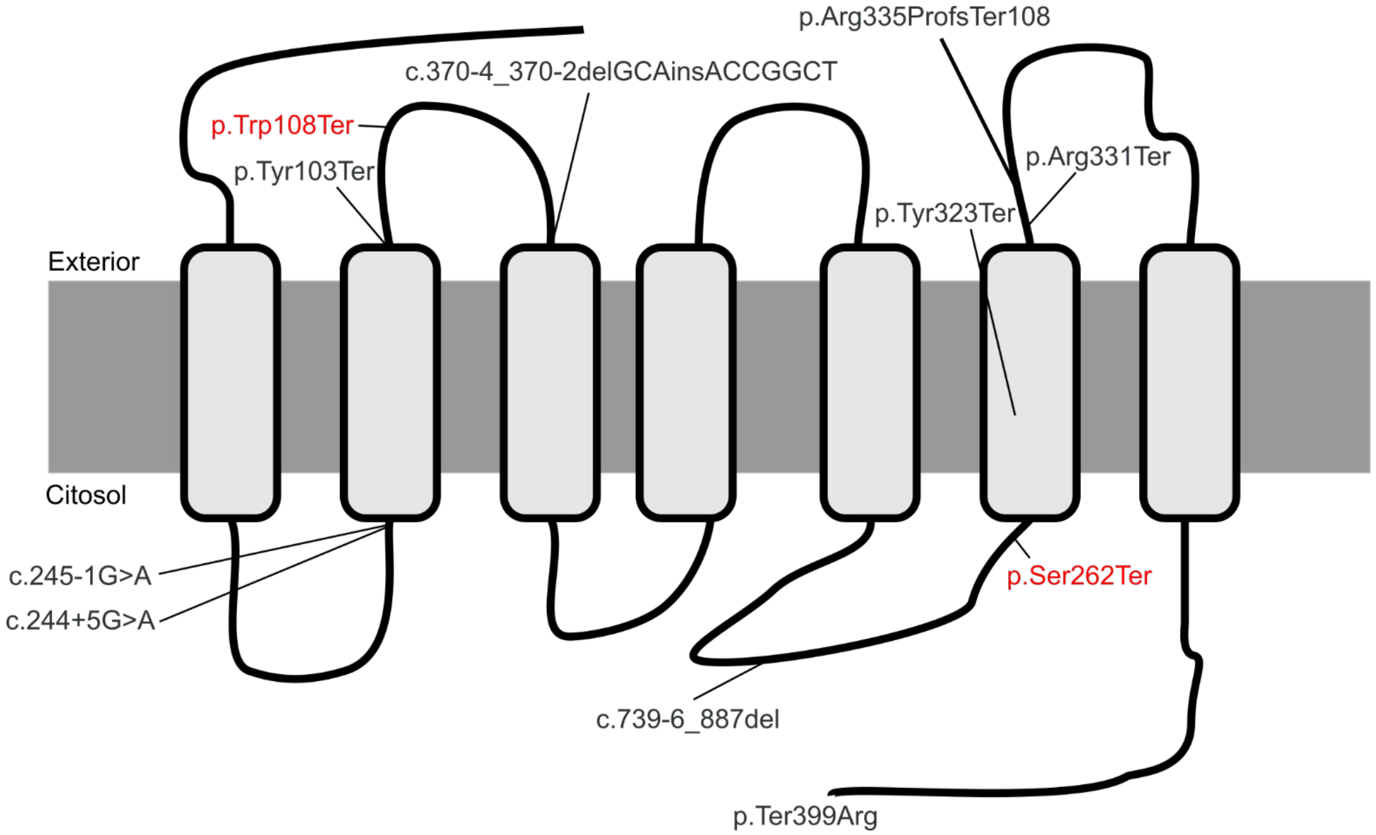
p.Arg335ProfsTer108
c.370-4_370-2delGCAinsACCGGCT
p.Trp108Ter
p.Tyr103Ter
p.Arg331Ter
p.Tyr323Ter
Exterior
Citosol
c.245-1G>A
c.244+5G>A
p.Ser262Ter
c.739-6_887del
p.Ter399Arg

Known CHH genes	Transcript RefSeq ID	Ref.
ANOS1	NM_000216	Bick, D et al. 1989
CHD7	NM_017780	Ogata, T. et al. 2006
FGF8	NM_033163	Falardeau, J. et al. 2008
FGFR1	NM_023110	Dode, C. et al. 2003
GNRH1	NM_000825	Bouligand, J. et al. 2009
GNRHR	NM_000406	de Roux, N. et al. 1997
KISS1	NM_002256	Topaloglu, A. K. et al. 2012
KISS1R	NM_032551	de Roux, Nicolas et al. 2003
NSMF	NM_001130969	Miura, Kiyonori et al. 2004
PROK2	NM_001126128	Dode, C. et al. 2006
PROKR2	NM_144773	Dode, C. et al. 2006
SEMA3A	NM_006080	Hanchate, N. K. et al. 2012
SOX10	NM_006941	Pingault, V. et al. 2013
TAC3	NM_013251	Topaloglu, A. K. et al. 2009
TACR3	NM_001059	Topaloglu, A. K. et al. 2009
WDR11	NM_018117	Kim, H. G. et al. 2010
AMH	NM_000479	Malone, Samuel Andrew et al. 2019
AMHR2	NM_020547	Malone, Samuel Andrew et al. 2019
AXL	NM_021913	Salian-Mehta, S et al. 2014
CCDC141	NM_173648	Hutchins, B Ian et al. 2016
DCC	NM_005215	Bouilly, Justine et al. 2018
DMXL2	NM_001174116	Tata, Brooke et al. 2014
DUSP6	NM_001946	Miraoui, Hichem et al. 2013
FEZF1	NM_001024613	Kotan, L. D. et al. 2014
FGF17	NM_003867	Miraoui, Hichem et al. 2013
FSHB	NM_001382289	Valdes-Socin, H et al. 2010
HS6ST1	NM_004807	Tornberg, J. et al. 2011
IL17RD	NM_017563	Miraoui, H. et al. 2013
KLB	NM_175737	Xu, Cheng et al. 2017
LEP	NM_000230	Strobel, A et al. 1998
LEPR	NM_002303	Clément, K et al. 1998
LHB	NM_000894	Valdes-Socin, Hernán et al. 2004
NDNF	NM_024574	Messina, Andrea et al. 2020
NOS1	NM_000620	Chachlaki, Konstantina et al. 2022
NR0B1	NM_000475	Muscatelli, F et al. 1994
NTN1	NM_004822	Bouilly, Justine et al. 2018
OTUD4	NM_001366057	Margolin, David H et al. 2013
PCSK1	NM_000439	O'Rahilly, S et al. 1995
PLXNA1	NM_032242	Kotan, Leman Damla et al. 2021
PNPLA6	NM_001166111	Topaloglu, A Kemal et al. 2014
POLR3A	NM_007055	Daoud, Hussein et al. 2013
POLR3B	NM_018082	Daoud, Hussein et al. 2013
RNF216	NM_207111	Margolin, David H et al. 2013
SEMA3E	NM_012431	Cariboni, Anna et al. 2015
SMCHD1	NM_015295	Shaw, Natalie D et al.
SOX2	NM_003106	Kelberman, Daniel et al. 2006
STUB1	NM_005861	Shi, Chang-He et al. 2014
TUBB3	NM_006086	Chew, Sheena et al. 2013
CHL1	NM_006614	Chen, Y et al. 2021
CPE	NM_001873	Alsters, Suzanne I M et al. 2015; Durmaz, Asude et al. 2021
DCAF17	NM_025000	Ali, R H et al. 2016; Fozia, Fozia et al. 2022
DLG2	NM_001142699	Jee, Youn Hee et al. 2020; Turan, Ihsan et al. 2021
LGR4	NM_018490	Mancini, Alessandra et al. 2020
CADM1 (NECL2)	NM_001301043	Tang, Ruiyi et al. 2020
NHLH2	NM_005599	Topaloglu, A Kemal et al. 2022
NRP2	NM_201266	Marcos, Séverine et al. 2017; Men, Meichao et al. 2021
NRP1	NM_003873	Marcos, Séverine et al. 2017; Men, Meichao et al. 2021
PLXNA3	NM_017514	Kotan, Leman Damla et al. 2021
POLA1	NM_001330360	Endrakanti, Mounika et al. 2021; Van Esch, Hilde et al. 2019
PRDM13	NM_021620	Whittaker, Danielle E et al. 2021
RAB3GAP2	NM_012414	Xu, Wanxue et al. 2020
SEMA3F	NM_004186	Kotan, Leman Damla et al. 2021
SEMA3G	NM_020163	Oleari, Roberto et al. 2021
SPRY4	NM_001127496	Miraoui, Hichem et al. 2013
ZNF462	NM_021224	Iivonen, Anna-Pauliina et al. 2021
